# Supplementary material for: Collective patterns of social diffusion are shaped by individual inertia and trend-seeking
Source: Nat Commun. 2021 Sep 29;12:5698. doi: 10.1038/s41467-021-25953-1 (PMC8481279; doi:10.1038/s41467-021-25953-1)
Supplement: Supplementary file 1 — Supplementary Information [file 41467_2021_25953_MOESM1_ESM.pdf]

Supplementary Information:
Collective patterns of social diffusion are shaped by individual inertia and trend-seeking

Mengbin Ye[1,2,*], Lorenzo Zino[2], Žan Mlakar[3], Jan Willem Bolderdijk[3], Hans Risselada[3], Bob M. Fennis[3], Ming Cao[2]

[1] *School of Electrical Engineering, Computing and Mathematical Sciences, Curtin University, Perth 6102, Australia*
[2] *Faculty of Science and Engineering, University of Groningen, 9747 AG Groningen, Netherlands*
[3] *Faculty of Economics and Business, University of Groningen, 9747 AG Groningen, Netherlands*

**Correspondence should be addressed to: mengbin.ye@curtin.edu.au*

Supplementary Notes **3**

Supplementary Note 1: Details and analysis of the experimental data 3

Supplementary Note 2: Statistical analysis of the experimental data 6

Supplementary Note 3: Example of the application of the classification method 8

Supplementary Note 4: Robustness of the diffusion phenomena 9

Supplementary Note 5: Comparison with different models for diffusion 11

Supplementary Note 6: Role of inertia and trend-seeking in the ABM 14

Supplementary Note 7: Explosive diffusion and delayed take-off in real-world examples 16

Supplementary Note 8: Simulation code 18

Supplementary Figures **19**

Supplementary Figure 1: Screenshots from selected pages in the game 19

Supplementary Figure 2: Experimental results showing group level diffusion over time . 20

Supplementary Figure 3: Monte Carlo simulations of the proposed model with the set of parameters "Second best fit with 16 trials", with 25% committed minority 21

Supplementary Figure 4: Monte Carlo simulations of the proposed model with the set of parameters "Best fit with all 20 trials", with 25% committed minority 22

Supplementary Figure 5: Monte Carlo simulations of the proposed model with the set of parameters "Best fit with 16 trials", with 17% committed minority 23

Supplementary Figure 6: Monte Carlo simulations of the proposed model with the set of parameters “Best fit with 16 trials”, with 33% committed minority 24

Supplementary Figure 7: Monte Carlo estimation of the switching rate for different fractions of explorers (ρ_e) 25

Supplementary Figure 8: Velocity plots of the proposed model 25

Supplementary Figure 9: Monte Carlo simulations of the proposed model with 25% of committed minority, with different definitions of the take-off time ($\bar{T}$) 26

Supplementary Figure 10: Monte Carlo simulations of the proposed model with 33% of committed minority, with different definitions of the take-off time ($\bar{T}$) 27

Supplementary Figure 11: Monte Carlo simulations of the proposed model with 17% of committed minority, with different definitions of the take-off time ($\bar{T}$) 28

Supplementary Figure 12: Comparison of the proposed decision-making model with a standard coordination game 29

Supplementary Figure 13: Diffusion simulated using an SIS epidemic model 29

Supplementary Figure 14: Sample simulations with only trend-seeking 30

Supplementary Figure 15: Sample simulations with only inertia 31

Supplementary Figure 18: Yearly use of the spelling “sólo” and “solo” in written Spanish for the adverb that means “only” 31

Supplementary Tables **32**

Supplementary Table 1: Summary of the results of the experiments at the macroscopic level 32

Supplementary Table 2: Data of experimental trial 17 and corresponding output of the classificator 32

Supplementary Table 3: Parameters of the model 33

Supplementary Table 4: Parameters of the models with only trend-seeking or only inertia 33

Supplementary References **34**

Supplementary Note 1: Details and analysis of the experimental data

Screenshots from selected pages of the game are presented in Supplementary Fig. 1, while Supplementary Fig. 2 extends Fig. 1a in the main article, plotting the temporal evolution of the diffusion process for each trial separately. The summary of the experimental results at the macroscopic (group) level are reported in Supplementary Table 1.

In the following, we provide several remarks on the experimental data to identify several key aspects which require careful consideration when we use the experimental data to parametrise the model.

Dropouts

Due to the online nature of the experiment, a number of trials saw recruits who registered but did not complete the instruction tasks; we refer to such recruits as "dropouts". Because the trials started immediately after the required number of recruits were signed on, it was not possible to delay the start of the trial to enrol further recruits to replace the dropouts. In order to maintain the same number of 12 players in each game, each dropout was replaced by a computer bot. These "dropout" bots, not to be confused with the committed minority bots, were programmed to always select the strategy that is the majority strategy in the current round and to select the status quo strategy if an equal amount of players chose each of the two strategies. Thus, the dropouts had a minimal impact on shaping the adoption of the alternative strategy, since the bots simply followed the majority and therefore were not promoting any particular strategy. Importantly, participants continued to see the strategy distribution of 11 other players in the game per round.

We stress that the dropouts did not participate in the trial in any way. A total of 32 dropouts were recorded over the 20 trials involving 180 recruited individuals (17.8% dropout rate), meaning 148 participants actually played the game. The maximum number of dropouts in a single trial was 4 (out of 10). Number of dropouts for each trial is summarised in Supplementary Table 1.

Missed Rounds

During the game itself, each round had a time limit of 20 seconds within which a participant was required to select between the two strategies. The game was programmed so that if a participant failed to select a strategy for a given round, then this was recorded, and, for the given round, the participant's response was given by the bot programmed in the same way as the "dropout" bots. That is, the participant's response was automatically selected to be the strategy that was selected by the majority in that given round, or to be the status quo strategy in case an equal amount of participants chose each of the two strategies.

Of the 148 participants, only 7 participants missed more than 20% of the round responses; we measure the percentage of missed round responses since missing 4 rounds in a trial that lasts 8 rounds is more significant than missing 4 rounds in a trial that lasts 24 rounds. These 7 partici-

pants were found in 5 of the trials, and are reported in Supplementary Table 1 under the “Missing Rounds” column. We presume that the reasons for missing rounds come down to being distracted by other activities occurring in the physical proximity of the participant. One participant even directly reported such an occurrence in the post-experimental feedback section: “Sorry for missing a few of the rounds! I had to make sure that my dog didn’t attack the mailman ;)”.

Participants with multiple missing round responses may impact the overall game in ways which cannot be easily characterised. “Dropout” bots aim to have a minimal impact by simply encouraging coordination (without being biased to the alternative or status quo strategy). Nonetheless, having too many responses determined by dropout bots for a given trial could be an issue. As we discuss in the Methods of the main article, we exclude trials in which too many participants missed rounds or were dropouts. Subsequent to this, we performed a robustness check by performing the same parametrisation and simulation study with all the trials included (see Supplementary Note 4).

Stubborn Participants

In 3 of the 20 trials, a single stubborn participant was observed in the group, as reported in Supplementary Table 1. A stubborn participant (not a computer bot) played the status quo strategy for every single round of the game, even when all other players (human participants and computer bots) were selecting the alternative strategy for multiple consecutive rounds. Thus, such a stubborn participant prevented the reaching of consensus; in a situation where all players but one participant were consistently selecting the alternative strategy for consecutive rounds, a rational participant would also switch in order to maximise his or her reward, whereas not switching would cause the group reward to be lost.

In 2 of these 3 trials, the stubborn participant was able to reverse the diffusion of the alternative, which can be seen in Supplementary Figs. 2n and 2p. In particular, we see that in each trial, a diffusion quickly takes off and all players except the stubborn participant rapidly converge to the alternative strategy, at the round marked by the dotted black line. Thereafter, an overwhelming majority continue to select the alternative strategy for a significant number of rounds. Then, in two of the trials, a second social diffusion phenomenon occurs and all participants explosively switch back to the status quo strategy. In the third trial (Supplementary Fig. 2r), there is a partial switch back to the status quo, and the game ends with an approximate 50-50 split in the strategy.

The presence of such stubborn participants consistently selecting the status quo is an interesting phenomenon since they are acting in direct opposition to the committed minority bots. It is also interesting to witness their ability to reverse the diffusion of the alternative strategy promoted by the computer bots. These findings suggest interesting future work on the role of stubborn status quo players in the diffusion of an alternative. There is a minor adjustment to the data of these trials for the parametrisation process, which we detail in the sequel. Moreover, we omit such individuals from simulations in our agent-based model, reported in the section titled “Explosive diffusion and delayed take-off are shaped by inertia and trend-seeking” of the main article, even if one could easily set parameters for an agent to mimic a stubborn status quo player. We omit such individuals

because the focus of this work is on the role of behavioural mechanisms in social diffusion, not on stubborn status quo players.

Summary of the 180 recruits

Overall, 32 of the 180 recruits were dropouts, resulting into 148 participants; 7 of them missed more than 20% of the rounds and 3 were stubborn, resulting into 138 regular participants (that is, 76.7% of the recruited party).

Example of an experimental trial

Finally, in order to better illustrate the functioning of the reward in the experiment, we extensively report the data of one experimental trial (in Supplementary Table 2), with the corresponding reward earned by each of the participants. Note that, since consensus was attained after 9 rounds, the total group reward for the group should have been equal to £10, if all the recruits participated to the experiment. However 2 of the 10 recruits dropped out. Hence, the total reward was equal to $\pounds 10 \times (8/10) = \pounds 8$. Such a group reward was then divided between the 8 participants, proportional to the number of times a participant selected the winning product (note that only active choices are valid, so a choice selected by a bot is never counted for splitting the reward). In this trial,

- player 8 selected the winning product once;
- players 4 and 10 twice;
- players 1 and 7 did it 3 times;
- players 2 and 9 did it 4 times; and
- player 6 selected the winning product 5 times

Hence, the group reward split among to the players equal to £0.33, £0.67, £1.00, £1.33 and £1.66, respectively. The total reward (which includes the base reward for participation) earned by each participant is reported in Supplementary Table 2.

Supplementary Note 2: Statistical analysis of the experimental data

Regression analysis

To support the presence of inertia and trend-seeking in the experimental data and illustrate their role on the individual-level behavioural patterns observed in the experimental data, we studied the data at the individual-level by employing a linear panel data regression analysis with a fixed effects estimator. The fixed effects estimator allowed us to control for any between-subjects variation in our data, thus exploiting only the over-time variation[1]. In other words, this approach ensures that any effects we discover can be attributed only to a participant reacting to something occurring in the earlier rounds of the game, and not to any differences between the participants.

Using this approach, we regressed the strategy that an individual chooses in a given round (hereafter termed choice in current round) on i) the proportion of others in their group who choose one strategy (hereafter termed adopters in previous round), ii) the strategy they chose in the previous round (hereafter termed choice in previous round), and iii) the proportion of others in their group who switched from one strategy to the other between two rounds earlier and the round prior (hereafter termed group trend). While choice in current round and choice in previous round are binary variables taking values in $\{0, 1\}$, adopters in previous round is a continuous variable ranging from 0 (if all other players choose the status quo in the previous round) to $+1$ (if all the other players choose the alternative), and group trend is a continuous variable ranging from -1 (if all other players switched from the alternative to status quo) to $+1$ (if the opposite occurs). We remark that the regression parameter associated with adopters in previous round captures the role of social coordination in our sample, the one associated with choice in previous round captures the role of inertia in our sample, whereas the one associated with group trend captures the role of trend-seeking in our sample.

The results of our regression analysis, which is statistically significant (F-score $= 494.66$, $p < 0.001$), are reported in Table 1 in the main article. From this analysis, we observed that adopters in previous round, choice in previous round, and group trend explains 46% of the over-time variance in the individual's choice in current round ($R^2 = 0.46$), and that all the three predictors are statistically significant ($p < 0.001$ for all the three predictors). In particular, from the regression parameters identified by our analysis, we observed that if an individual has chosen a given strategy in the previous round, the probability that they will choose it again in the current round increases by 36%. Moreover, if the whole group except the given individual switched strategy in the previous round (generating thus a trend), then the probability that the considered individual will also choose the trending strategy in the current round increases by 31%. In summary, the regression analysis examining the experimental data at the individual level provided support for the presence and the significance of inertia and trend-seeking (besides social coordination) in the individual-level decisions that participants made during our experimental game and, consequently, in creating the shape of the diffusion patterns observed. This finding thus provides us with further motivation to include inertia and trend-seeking —together with social coordination— as the key mechanisms underlying each agent's decisions in the ABM.

Inertia

We confirmed the results of the regression analysis by studying the two additional tests on the individual-level data, focusing separately on inertia and trend-seeking. As inertia is concerned, we performed a Wald–Wolfowitz runs test[2]. Specifically, from the time series of individuals' decisions (see, e.g., Supplementary Table 2), we consistently observed statistically significant patterns of consecutive rounds in which the same individual selected the same decision. In fact, the Wald–Wolfowitz runs test, provided a z-value equal to -25.51, with $p < 0.0001$. This observation, together with the results of the regression analysis described above, suggests that inertia may play an important role in the individual's decision-making process, in agreement with existing literature on inertia and status quo bias[3,4].

Trend-seeking

Next, we analysed individual-level decision making in a scenario in which a trend was present, that is, when the fraction of adopters of the two strategies among the rest of the participants has changed in the previous time step; thus, one strategy has an increasing trend and one has a decreasing trend. Across the entire experimental data, individuals switched their strategy 179 times when in such a scenario, 137 times changing to the strategy with increasing trend, while only changing to the strategy with decreasing trend 42 times. In particular, there were 17 instances where an individual was faced with two strategies at 50%–50%, with a trend present, and subsequently changed strategy in the next round. Here, social coordination would suggest that an individual would favour the two strategies with equal probability, while inertia would reduce the switching probability uniformly across the two strategies. Hence, neglecting the trend, we would have expected to observe switches in both direction with the same frequency. However, in 14 of the 17 instances, the individual selected the strategy with increasing trend. Such a bias is statistically significant, as confirmed by a binomial test[2], with $p \approx 0.005$. This corroborates the results of the regression analysis, confirming that the change in strategy distribution over time can impact the decision-making process, inline with existing literature such as the work on dynamic norms[5–7], supporting its inclusion in our ABM

Supplementary Note 3: Example of the application of the classification method

Supplementary Table 2 shows one experimental trial (trial 17, illustrated in Supplementary Fig. 2q) and the associated output of the classification method, which was described in the Methods section of the main article.

Supplementary Note 4: Robustness of the diffusion phenomena

Additional simulations were conducted to verify that the three key features of the diffusion process (delayed take-off, explosive diffusion, and moderate and heterogeneous switching activity) are intrinsic to the proposed model and robust against variations in the parameters. To this aim, we investigated the properties of the diffusion process parametrised using two other sets of parameters, corresponding to the second best fit of the experimental data and the best fit without removing the 4 trials with many irregular players, which are reported in Supplementary Table 3.

Then, we repeated the analysis, with the set of parameters used in the main paper, for fractions of committed minority comprising 17% and 33% of the population, which correspond to the other two experimental settings. The results are reported over the next several pages and Supplementary Figs. 3–7. Detailed explanations of our approach and findings are found in the relevant captions.

The results of our numerical simulations provided evidence that the three key features of the diffusion process are intrinsic to the proposed model and robust against variations in the parameters and in the number of committed minority. Interestingly, by comparing Supplementary Figs. 5a and 6a, we found that the number of committed minority may play an important role in determining the take-off time. Specifically, we observed that when the committed minority are reduced, the delay in the take-off time seems to depend on the population size n, exhibiting a phase transition between sub-linear and super-linear growth with respect to n, depending on the number of explorers. If the committed minority are increased, then the take-off time seems to be independent of the population size, even in the absence of explorers. Surprisingly, this phase transition seems to appear when the fraction of committed minority is about 25%, which is consistent with the critical mass threshold for changes in social conventions, empirically identified in Centola et al.[8].

In the main paper, we defined $\bar{T}$ as the time needed for the diffusion process to take-off, identified as the last time step before the diffusion in which no more than 40% of the players (including the bots) selected the alternative ($\frac{1}{n}\sum_{v\in\mathcal{V}} x_v(t) = 0.4$). Here, we provide additional analyses and simulations to motivate and support our choice of setting such a threshold value at 0.4. First, we show a velocity plot in Supplementary Fig. 8, which reports the change in the fraction of adopters of the alternative $\Delta z(t) := \frac{1}{n}\sum_{v\in\mathcal{V}} x_v(t+1) - \frac{1}{n}\sum_{v\in\mathcal{V}} x_v(t)$, as a function of the current fraction of adopters $z(t) := \frac{1}{n}\sum_{v\in\mathcal{V}} x_v(t)$, that is, $\Delta z(z(t)) = z(t+1) - z(t)$. We average over $10,000$ independent simulations with $n = 200$, with 25% of committed minority. Panels (a) and (b) show fractions of explorers $\rho_\mathrm{e} = 0.2$ and $\rho_\mathrm{e} = 0.6$, respectively. The results of our Monte Carlo simulations clearly indicate that the diffusion process takes off when the fraction of adopters is close to 0.4. In fact, once the fraction of adopters of the alternative exceeds a threshold of about 0.4, then the velocity of the diffusion process quickly grows.

Finally, we run a robustness check on the definition of the take-off time. Specifically, in Supplementary Figs. 9–11 we show the take-off time $\bar{T}$ and the transition time ΔT for the three different scenarios (17%, 25%, and 33% of committed minority) by decreasing and increasing the threshold from 0.4 to 0.35 (panels a and b) and 0.45 (panels c and d), respectively. All the results obtained are qualitatively consistent with our findings with the threshold at 0.4. However, we men-

tion that setting the threshold at 0.35 might be misleading in the scenario with 33% of committed minority, as the take-off time does not properly capture the moment at which the diffusion process exists the meta-stable state around the status quo (due to the stochasticity of the process).

Supplementary Note 5: Comparison with different models for diffusion

In this Supplementary Note, we compare the proposed decision-making model with other models from the diffusion literature. In the main article (Fig. 3), we have compared our model with a standard decision-making process based on a coordination game, that is, in the absence of the second and third terms in Equations (2a) and (2b) in the main article, that account for inertia and trend-seeking. To account for the heterogeneity in the population, we set up the coordination game by setting different levels of rationality β_v for explorers and non-explorers, and calibrated it with our experimental data using the same procedure employed for our model, detailed in the Methods. We obtained $\beta_{\mathrm{e}} = 4.8$ for the explorers and $\beta_{\mathrm{f}} = 19.7$ for the non-explorers. First of all, we observe that such a parametrisation seems to be sub-optimal in terms of the cost C defined in Equation (7) in the Methods. In fact, considering only coordination (even with heterogeneous rationality parameters) causes an increase in the value of the functional C that corresponds to the best fit of the data by 8140%, compared to the value of C when inertia and trend-seeking are included. Additional simulations, illustrating the behaviour of a coordination game when all the individuals have high ($\rho_{\mathrm{e}} = 0$) or low ($\rho_{\mathrm{e}} = 1$) rationality are reported in Supplementary Fig. 12, respectively, to further corroborate our observations.

The results of our analysis confirmed the importance of the inertia and trend-seeking terms evidenced in the social psychology literature and showed that they are key for the diffusion process to exhibit all the microscopic and macroscopic features and the variety of behaviours observed in the experiment and in real-world data. In the following, we discuss other two modelling frameworks that have been used to capture diffusion processes (epidemic-like models[9], and linear threshold models[10]), showing their main limitations in modelling of social diffusion.

Epidemic-like models

Here, we report the simulations of an epidemic-based diffusion process. We implemented the strategy revision process by means of a stochastic susceptible–infected–susceptible (SIS) model, which is one of the most adopted models in epidemiology, and has been used to model spreading processes on complex networks[9]. According to the SIS model, each player $v \in \mathcal{V}$ is characterised by two parameters: the infection probability $\lambda_v \in [0,1]$ and the recovery probability $\mu_v \in [0,1]$. Then, the probability for player v of switching strategy from 0 (Susceptible) to 1 (Infected) at time $t+1$ is given by

$$\mathbb{P}[x_v(t+1) = 1 \,|\, x_v(t) = 0] = 1 - (1-\lambda_v)^{\sum_{w \in \mathcal{V} \setminus \{v\}} x_w(t)} \,,$$

and the probability for player v of switching strategy from 1 to 0 at time $t+1$ is equal to

$$\mathbb{P}[x_v(t+1) = 0 \,|\, x_v(t) = 1] = \mu_v \,.$$

In the absence of any committed minority, epidemic models often exhibit a threshold phenomenon: if the infection probabilities λ_v are small with respect to the recovery probabilities μ_v, then the contagious disease effectively never diffuses in the population. Otherwise, if the infection probability

is sufficiently high, then an epidemic outbreak occurs and the disease spreads immediately, i.e. with no delay, and rapidly through the population, reaching an endemic equilibrium. The infection and recovery probabilities that determine a threshold at which the system switches from one regime to the other is typically refer to as the epidemic threshold of the model Supplementary Equation ().

The diffusion process is modelled similar to the one proposed in the main article, in subsection "Diffusion driven by a committed minority". Specifically, we identify susceptible agents as those that choose the status quo strategy, while infected individuals adopt the alternative. We consider a population with all players that start from $x_v(0) = 0$. Then, we introduce a set of committed minority $\mathcal{C}$ that stubbornly play strategy 1. This is obtained by setting $x_c(0) = 1$ and $\mu_c = 0$, for all $c \in \mathcal{C}$. For the other individuals, we consider an homogeneous scenario, in which $\mu_v = \mu$ and $\lambda_v = \lambda$, for all $v \in \mathcal{V} \setminus \mathcal{C}$. In this scenario, the asymptotic behaviour of the system is determined by the ratio λ/μ, while the magnitude of the two parameters determines the duration of the transient phase.

We ran the simulations with population size equal to $n = 200$, $|\mathcal{C}| = 0.25n$ committed minority, and different values of the parameters λ and μ (all above the epidemic threshold that guarantees diffusion to occur, that is, $\lambda > \mu/(n-1)$). The simulation outcomes are reported in Supplementary Fig. 13.

We observe that the diffusion process always has an immediate take-off. Hence, the SI model can produce i) highly explosive transition with no (or very short) delay, ii) moderate explosive transition with moderate delay, or iii) almost linear growth in which the transition time ΔT and the diffusion time T^* are always comparable. The blue and red curves illustrate extreme outcomes of i) and iii), respectively. Hence, it seems that the SIS model cannot simultaneously produce a long delay with an explosive transition.

Linear Threshold model

The linear threshold model (LTM) has been proposed as a deterministic agent-based model to reproduce and study cascading phenomena[10,11]. In the LTM, each agent v is characterised by a threshold $\Theta_v \in [0, 1]$. Then, agent v updates its strategy according to the following law:

$$x_v(t+1) = \begin{cases} 1, & \text{if} \quad \dfrac{1}{n-1} \displaystyle\sum_{w \in \mathcal{V} \setminus \{v\}} x_w(t) \geq \Theta_v, \\ 0, & \text{else.} \end{cases} \tag{1}$$

Diffusion processes modelled by LTMs exhibit a monotonic behaviour, that is, $x_v(t+1) \geq x_v(t)$ for all v. In other words, each agent can only either switch to 1 at a certain time and keep playing 1 from that time on, or keep playing 0 for all the duration of the process. This also implies the diffusion curve at the group level is monotonically nondecreasing. This observation is not consistent with our empirical observations, where several non-monotonic trends have been observed (see, for instance, Supplementary Figs. 2a, 2b 2k and 2kl), and 63 of the 138 regular participants (i.e. more than 45%) did more than just one switch.

From a macroscopic point of view, the diffusion process is fully determined by Supplementary Equation (1) and, ultimately, on the distribution of the thresholds Θ_v. Hence, a delayed and explosive diffusion might be obtained by properly defining a heterogeneous distribution of thresholds. However, this requires an extremely large number of variables, which is in contrast with standard scientific principles based on Occam's razor. Another possibility to observe delayed and explosive diffusion is to introduce a network structure. However, the experimental results evidenced that the network structure is not necessary to observe delay and explosiveness, which were observed in our all-to-all experimental setup.

Supplementary Note 6: Role of inertia and trend-seeking in the ABM

In this Supplementary Note, we elucidate the role of inertia and trend-seeking, by examining the diffusion patterns that emerge when only one of the two mechanisms is present. To this aim, we consider an implementation of the model with inertia and social influence (i.e. without trend-seeking), and a separate implementation with only trend-seeking and social influence (thus with no inertia). In both the implementations, we use the same procedure for the calibration of the model as detailed in the Methods section of the main article, but set $r_\mathrm{e} = r_\mathrm{f} = 0$ and $k_\mathrm{e} = k_\mathrm{f} = 0$ for the implementation with no trend-seeking and no inertia, respectively. The parameters obtained are detailed in Supplementary Table 4.

Before discussing the individual-level and emergent behaviour of these models, it is worth noticing that both implementations are sub-optimal in terms of the cost C defined in Equation (7) in the Methods. In fact, removing inertia and trend-seeking causes an increase in the value of the functional C that corresponds to the best fit of the data by $17,136\%$ and 71%, respectively. This suggests that, in the absence of inertia, the model is essentially unable to capture the individual level switching rate data from the experiments.

Supplementary Fig. 14 shows the model with the trend-seeking mechanism, and with no inertia. Notice from Supplementary Table 4 that all individuals have a high rationality, $\beta = 14$; if there is no trend at a given time instant, an individual with such a high β playing the status quo has a probability smaller than 0.1% of joining a 25% committed minority. Moreover, non-explorers have $r_\mathrm{f} = 0$, which implies that even in the presence of trend, they will (almost) never join a minority. As a consequence of such an high rationality, at the macroscopic level, no diffusion is observed. At the microscopic level, a mildly heterogeneous switching rate distribution is observed, with two peaks. The peak around zero matches that of the empirical data (Fig. 1b in the main article) and corresponds to the activity of the non-explorers, while a second peak corresponding to the explorers increases as ρ_e increases (Supplementary Fig. 14b and 14c). However, the distribution of the switching rates seems to be concentrated around the peaks, not matching the high heterogeneity observed in the empirical data.

Supplementary Fig. 15 shows the model with the inertia mechanism, but with no trend-seeking. Notice that in order for the model parameters to match our empirical data, the parametrisation process identified the rationality to be much lower, $\beta = 5.8$, and explorers have almost no inertia, with $k_\mathrm{e} = 0.04$. At a microscopic level, the switching rate activity shows heterogeneity, with a distribution that closely resembles the empirical data from Fig. 1b in the main article (large peak around 0, and the remaining rates widely distributed). However, we see that the macroscopic diffusion patterns do not exhibit the same characteristic features of delayed take-off and explosive diffusion. In particular, the diffusion time T^* can be delayed by flattening the diffusion curve by reducing the number of explorers. Consequently, the delay in diffusion increases and the explosiveness of the process decreases. Thus, trend-seeking seems to be key to enable delayed explosive diffusion.

We can clearly see from Supplementary Figs. 14 and 15 that adding just one of the two behavioural mechanisms to the coordination mechanism is not sufficient for the model to display the

key characteristics of diffusion at both the macroscopic and microscopic level. This highlights that the interplay between the inertia and trend-seeking mechanisms is highly nontrivial, and contribute together to the overall dynamics. It is not straightforward to untangle their effects, and it suggests both mechanisms need to be considered in the agent-level dynamics, confirming and corroborating the findings of the regression analysis reported in Supplementary Note 2.

Supplementary Note 7: Explosive diffusion and delayed take-off in real-world examples

In this Supplementary Note, we report on three different examples of real-world diffusion processes that exhibit features observed in the proposed model, i.e., i) delayed take-off time and ii) explosive transition. We remark that there is little data available on the agent-level switching activities for the three examples, but it is not difficult to imagine that in the activities of the considered examples, an agent would be reluctant to switch often.

In particular, we consider i) the rejection of footbinding practice among Chinese women at the turn of the 20th century[12], and ii) the adoption of periphrastic "do" through different syntactic environments in English between the 15th and the 18th century[13], and iii) the diffusion of the spelling "sólo" instead of "solo" for the Spanish adverb meaning "only" in the second part of the 19th century[14].

We wish to note that we are not claiming that the model proposed in this work can be directly used to predict and capture the diffusion process of each of these real-world examples. The proposed model is still relatively minimalistic, whereas the real-world examples have a variety of exogenous and complicating factors. For instance, there are still debates ongoing as to the true driver of the ending of footbinding practices, and the success of the use of "sólo" (which was finally regulated in year 1956 by the Royal Spanish Academy) might be due to avoiding misunderstandings with the homographic adjective "solo". We aim only at highlighting that such real-world diffusion processes do exhibit the features that are consistently observed in our experimental data and in the proposed model.

Cessation of footbinding

The cessation of footbinding practice among Chinese women is a well-known and extensively studied paradigm shift in social conventions that occurred at the beginning of the 20th Century[12, 15]. In this scenario, the "status quo" strategy is footbound, and the alternative strategy is not footbound. A representation of such a process can be found in Figs. 7 and 8 of Brown & Satterthwaite-Phillips[12], showing the fraction of women interviewed that had their foot bound, from different parts of rural China. It is important to take into consideration the following points. First, because footbinding took place while the female was a young child and involved the application of a binding cloth over months or even years, the length of a single time-step is extremely large (at least greater than a year). Second, it is recorded that footbinding was the normative practice as far back as the Ming Dynasty (1368-1644), and that centralised efforts to abolish these practices date as far back as 1665 by the Manchu government[15]. However, it was not until the 1920s that women in Northern and Central China started abandoning such a practice, while (almost) full rejection is observed starting from the beginning of the 1940s. Given this context, one could argue that the alternative (not footbound) has both a long delay in the take-off time and the diffusion is explosive once diffusion process takes off.

Use of periphrastic “do”

The use of periphrastic “do” through different syntactic environments in English from 1390 to 1710[13]. The works of D. Danison[16] (page 278) and the data gathered by R. Vulanović[17] report three different temporal evolution for the use of “do” in negative questions (“do I read” vs “read I not”,), in negative declarative sentences (“I do not read” vs “I read not”) and in positive declarative sentences (“I read” vs “I do read”). The former had an explosive diffusion with a short delay, whereby the use of periphrastic “do” became widespread at the beginning of the 16th century. The use of “do” in negative declarative sentences, instead, had a delayed explosive adoption, whereby its used, even though already present in the 15th century, became popular only in the second part of the 17th century. Finally, no diffusion of the periphrastic “do” has been observed for positive declarative sentences.

Spelling “sólo” and “solo” in written Spanish

Despite the fact that its use was already recorded at the beginning of the year 1800 (starting point of the dataset), the curves depicted in Supplementary Fig. 16 show a delayed adoption of the spelling “sólo”, which started only after 1860. These graphs are generated using Google Ngram[18,19]. Note that the use of “sólo” quickly became dominant, despite its use only being regulated in 1956 by the Royal Spanish Academy. Finally, we observe that, after a “meta-stable” equilibrium that lasted for almost a century, in which “sólo” was the dominant choice, the spelling “solo” had an explosive diffusion in the past decade.

Supplementary Note 8: Simulation code

The code used in the simulations is written in MATLAB and has been deposited in the Zenodo database, under the accession code https://doi.org/10.5281/zenodo.5175151 using an MIT License for open-source availability[20]

The code consists in a loop that, with fixed model parameters (the number of agents n and of committed minority bots $|\mathcal{C}|$, the rationality β, and the coefficients b_v, k_v and r_v, for all $v \in \mathcal{V} \setminus \mathcal{C}$) and the initial condition $x(1) \in \{0,1\}^{|\mathcal{V} \setminus \mathcal{C}|}$, and initialised the time-index as $t = 1$, updates individuals' choices according to the following algorithm:

1. for each agent $v \in \mathcal{V} \setminus \mathcal{C}$, compute $\hat{x}_v(t)$ as defined in Equation (3) in the main article[1];
2. for each agent $v \in \mathcal{V} \setminus \mathcal{C}$, compute the payoff functions $\pi_v(1)$ and $\pi_v(0)$ as in Equation (2) in the main article;
3. for each agent $v \in \mathcal{V} \setminus \mathcal{C}$, compute the probability of adopting $+1$, defined in Equation (1) in the main article;
4. for each agent $v \in \mathcal{V} \setminus \mathcal{C}$, simulate a random variable $rand_v(t)$, uniformly distributed in $[0,1]$, independent of the other agents. If $rand_v(t)$ is less than or equal to the probability computed in step 3, then set $x_v(t+1) = +1$; otherwise, set $x_v(t+1) = 0$;
5. increase the time-index t by 1. If $t < T$, where T is the stopping time, the algorithm resumes to step 1, otherwise the algorithm stops.

The repository contains also a sample simulation of a diffusion process with the script used to generate it and all the scripts to generate the figures[20].

[1]For $t = 1$, set $\hat{x}_v(t) = 0$, for all $v \in \mathcal{V} \setminus \mathcal{C}$.

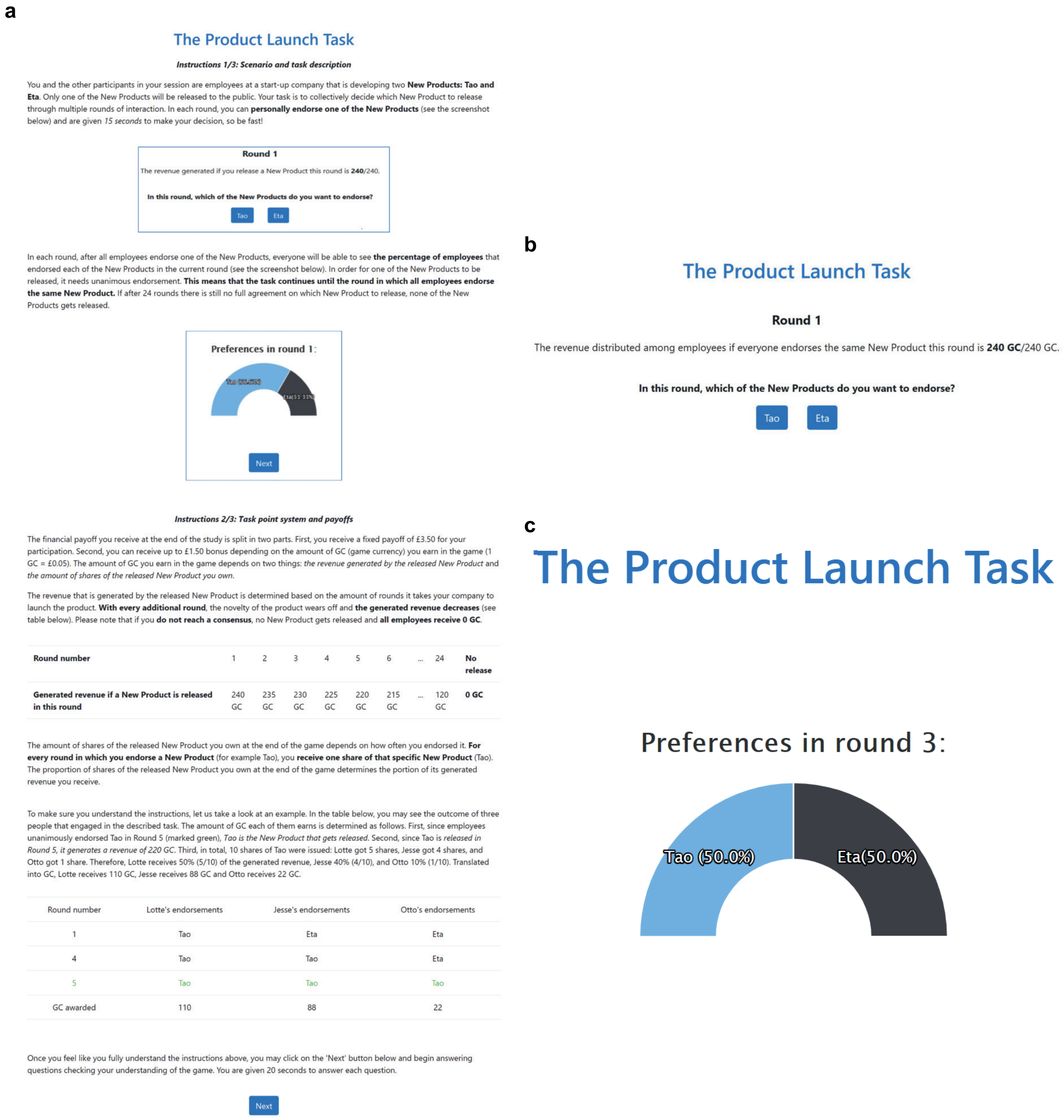

Supplementary Figure 1: **Screenshots from selected pages in the game.** **a** Instruction page. **b** Form through which participants are informed of the total group reward remaining and are asked to make their choice. **c** Then, after all players have selected a product (strategy) for a given round, each participant is shown the distribution of the choices of the other players (including all of the participants, committed minority bots and dropout bots).

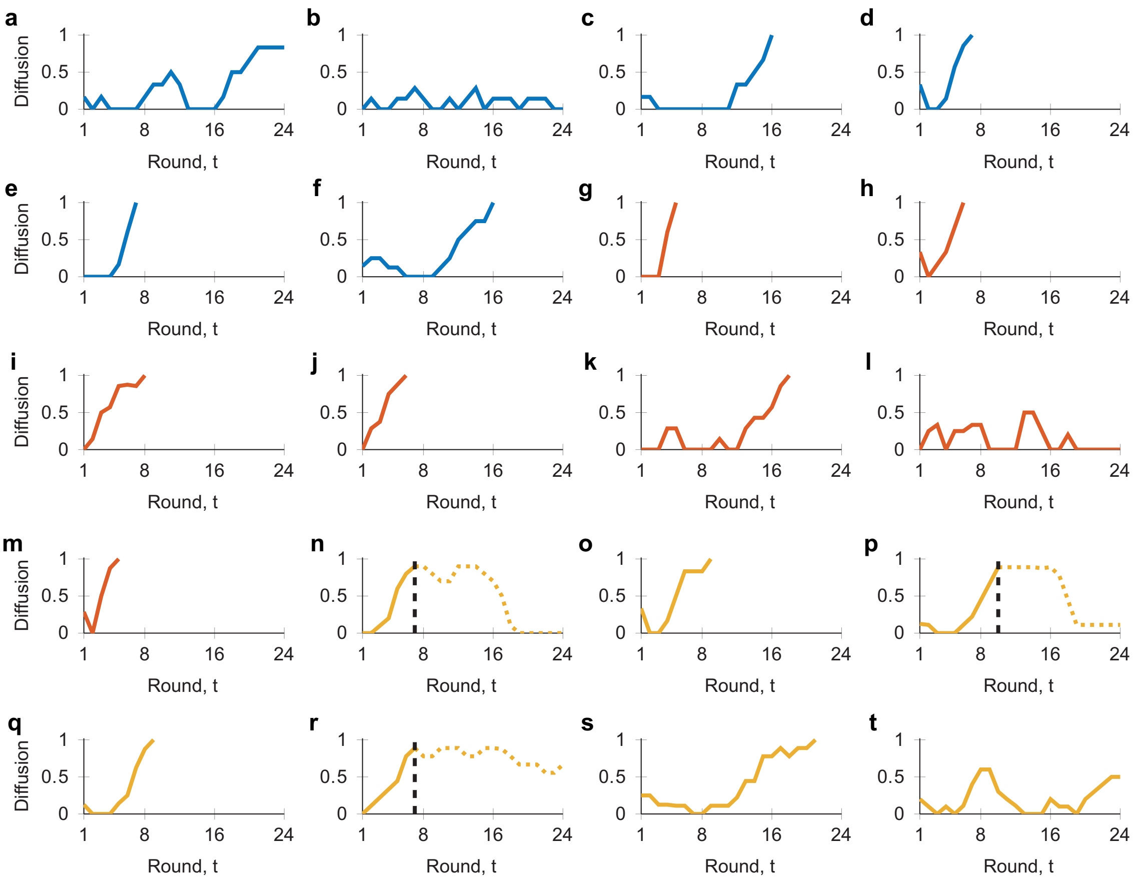

Supplementary Figure 2: **Experimental results showing group level diffusion over time**. Figures show the temporal evolution of the fraction of participants adopting the alternative strategy in the experiments (y-axis) 25% (panels **a**–**f**), 33% (panels **g**–**m**), and 17% (panels **n**–**t**) of committed minority. The trials in panels **n**, **p**, and **r** are the three trials with totally stubborn individuals. The vertical dashed line denotes the round in which the regular participants (i.e., excluding the stubborn individuals) reach consensus.

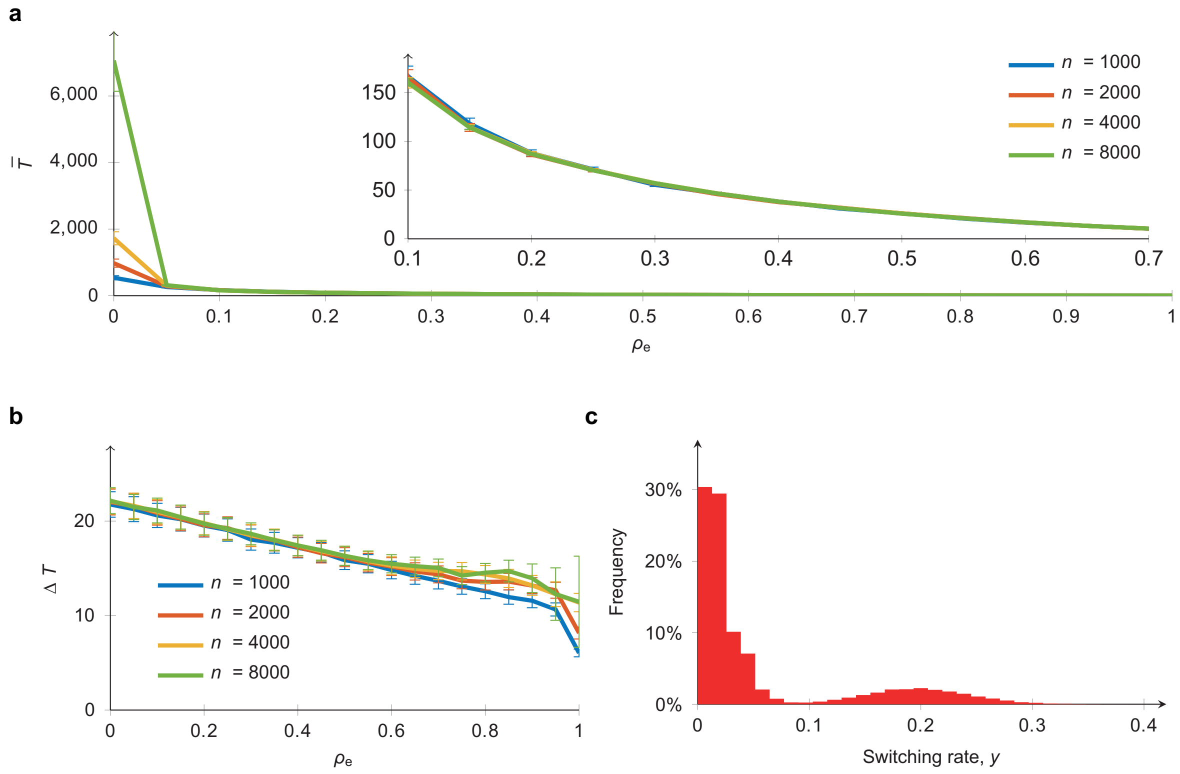

Supplementary Figure 3: **Monte Carlo simulations of the proposed model with the set of parameters "Second best fit with 16 trials", with 25% committed minority.** The quantities $\bar{T}$, ΔT, ρ_e and y are defined in the main article. The qualitative behaviour of the system coincides with the one observed with the set of parameters "Best fit with 16 trials", reported in the main paper. **a** There is always a non-negligible take-off time, which grows as fraction of explorers decreases. If there are explorers, then the take-off time seems to be independent on the population size n. However, for a population comprised only of non-explorers, the take-off time seems to grow greater than linearly in the population size. **b** The diffusion is always explosive, independently of the population size and it is mildly affected be the fraction of explorers in the population. **c** At the individual level, the simulations suggest that the switching rate is moderate and heterogeneously distributed. These are all consistent with the simulations reported in the main article.

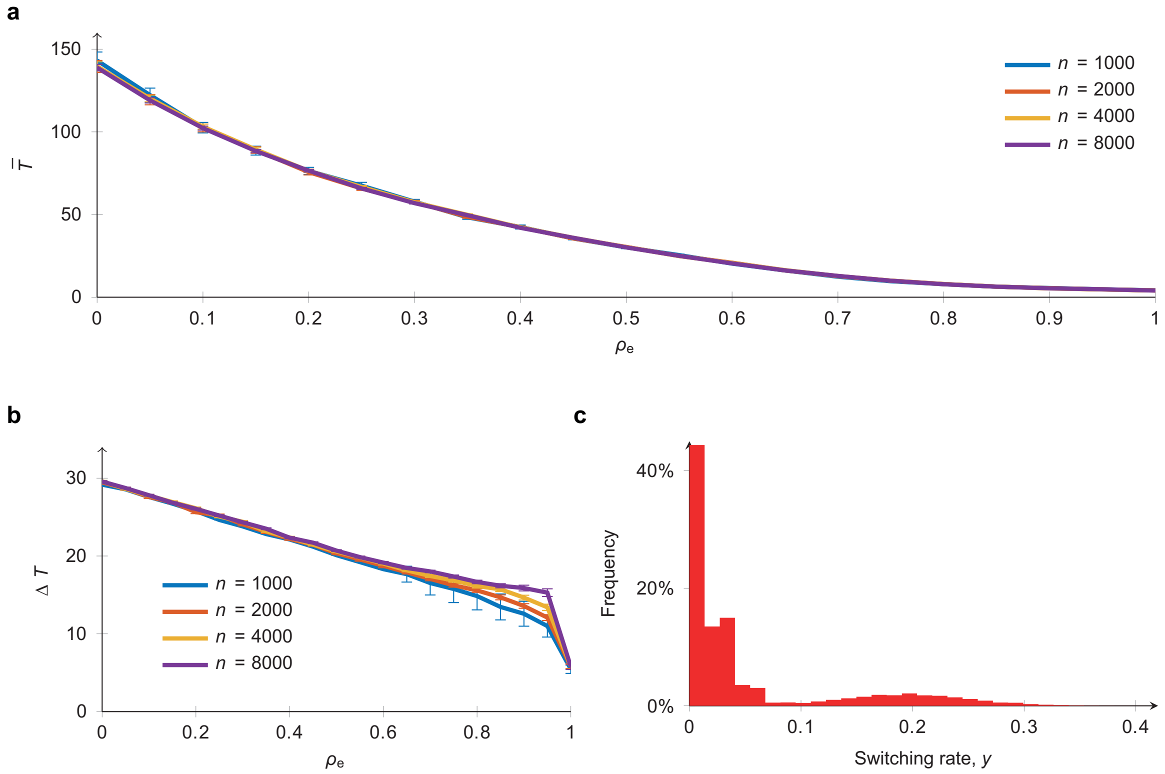

Supplementary Figure 4: **Monte Carlo simulations of the proposed model with the set of parameters "Best fit with all 20 trials", with 25% committed minority.** The quantities $\bar{T}$, ΔT, ρ_e and y are defined in the main article. The qualitative behaviour of the system is consistent with the one observed with the set of parameters used in the main paper. **a** There is always a non-negligible take-off time, which grows as the fraction of explorers decreases. Differently from the other two sets of parameters, here we do not observe the phase transition in the absence of explorers: if there are no explorers, the take-off time still seems to be independent of the population size. A possible explanation for this difference is that we have identified a lower level of rationality β, which may be due to the use of unreliable data caused by the presence of many irregular individuals in the population. Hence, individuals that are categorised as non-explorers, have a higher tendency to make random decisions, which seems to decrease the effect of inertia, and thus reducing the diffusion time. **b** The diffusion is always explosive, independently of the population size and it is mildly affected by the fraction of explorers in the population. **c** At the individual level, the simulations suggest that the switching rate is moderate and heterogeneously distributed.

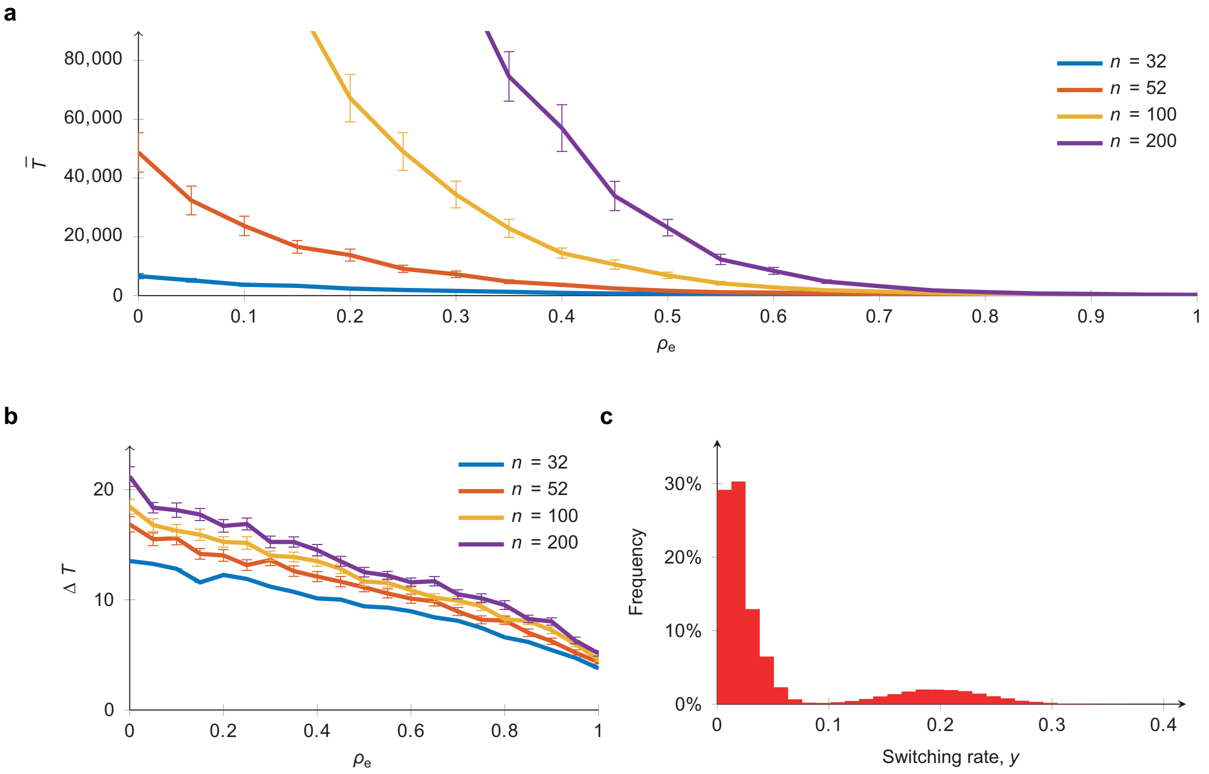

Supplementary Figure 5: **Monte Carlo simulations of the proposed model with the set of parameters "Best fit with 16 trials", with 17% of committed minority. a** The main difference with respect to the case with 25% of committed minority can be observed in the take-off time. Similar to the case with 25% of committed minority, the take-off is always delayed, and the delay grows as the fraction of explorers ρ_e decreases. However, in the presence of fewer committed minority, the delay in the take-off seems to depend significantly on the population size (while with 25% of committed minority, this happens only in the absence of explorers). Besides this difference, which is consistent with our findings in the main article (section "Key role of committed minority and explorers" of the main paper), the qualitative behaviour of the system is consistent with that observed with 25% of committed minority. **b** The diffusion is always explosive; it is mildly affected be the fraction of explorers in the population and it seems to be mildly affected by the population size n. Note that the diffusion time ΔT is small even when the fraction of explorers is such that the take-off time grows greater than linearly with the population size. **c** At the individual level, the simulations suggest that the switching rate is moderate and heterogeneous.

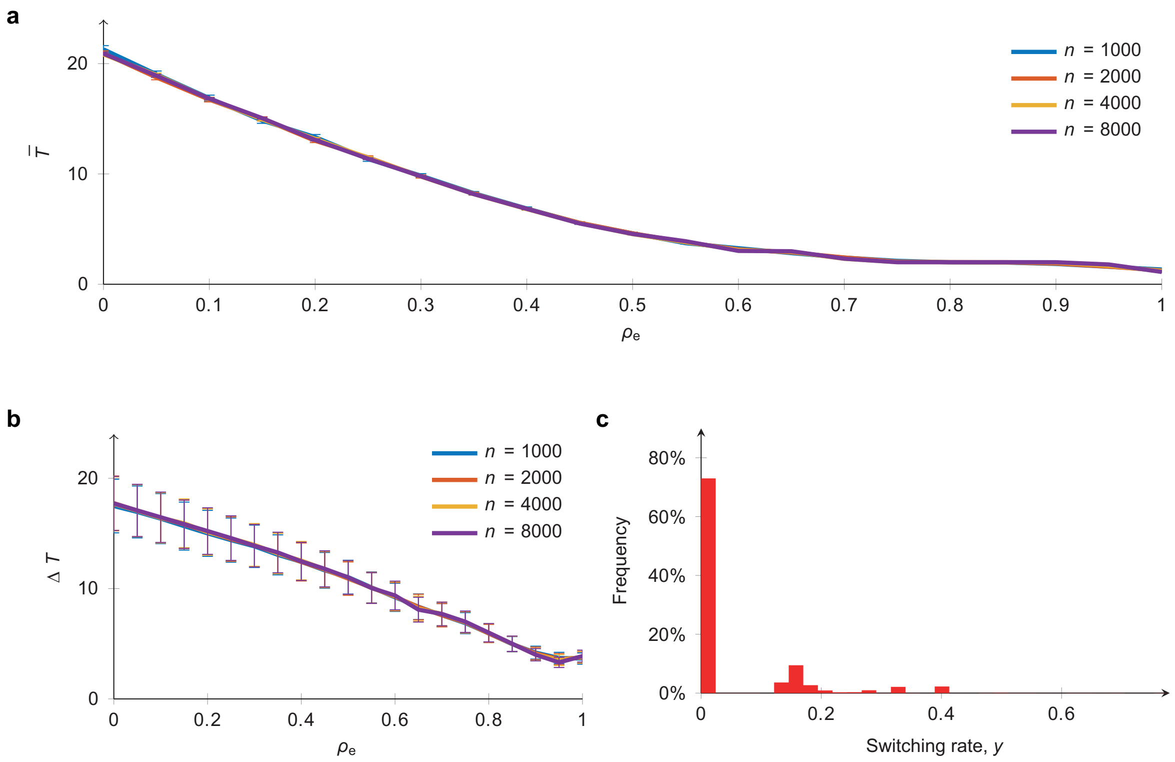

Supplementary Figure 6: **Monte Carlo simulations of the proposed model with the set of parameters "Best fit with 16 trials", with 33% of committed minority.** The quantities $\bar{T}$, ΔT, ρ_e and y are defined in the main article. The qualitative behaviour of the system is consistent with what is observed with 25% of committed minority. The main difference seems to be that, by increasing the number of committed minority, the delay in the take-off seems to be independent of the population size n, even in the absence of explorers. This is consistent with our findings in the section "Key Role of committed minority and Explorers" in the main paper.

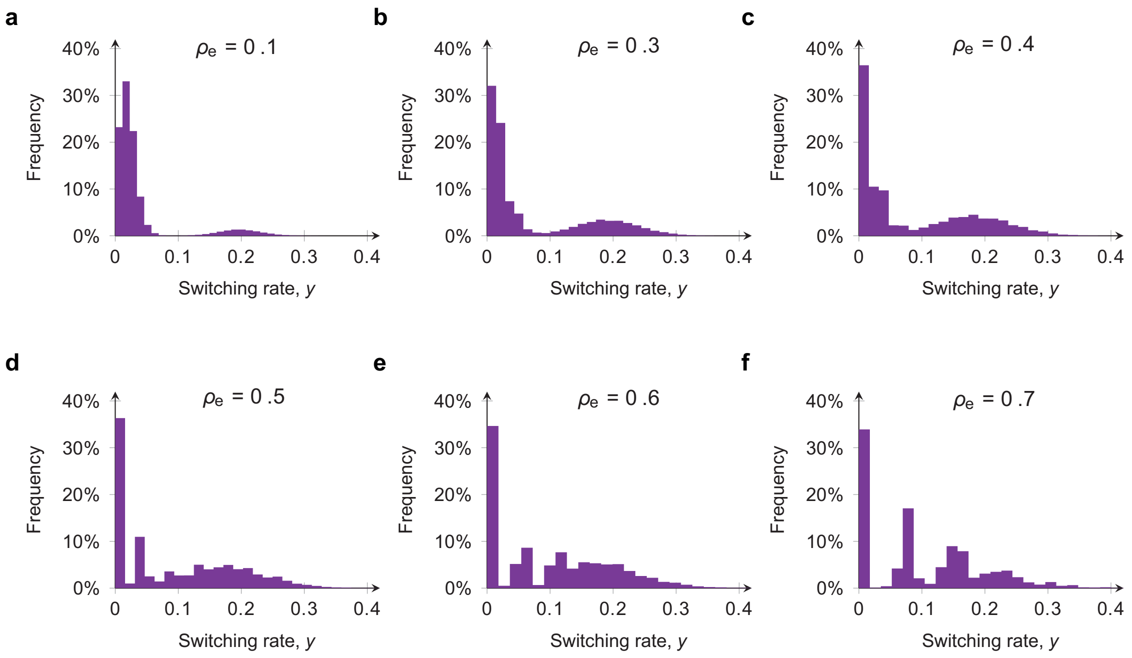

Supplementary Figure 7: **Monte Carlo estimation of the switching rate for different fractions of explorers (ρ_e).** Estimations are performed over 1000 independent simulations, for a population size $n = 1000$, with the set of parameters "Best fit with 16 trials", extending the results reported in Fig. 4c in the main article. All the simulations show a moderate and heterogeneous switching rate, with a peak concentrated close to 0 and the remaining rates widely distributed (and such a width increases as the fraction of explorers grows). The average number of switches is always moderate and increases with the number of explorers in the population, from one switch every 19 rounds (panel **a**), to once every 10 rounds (panel **f**).

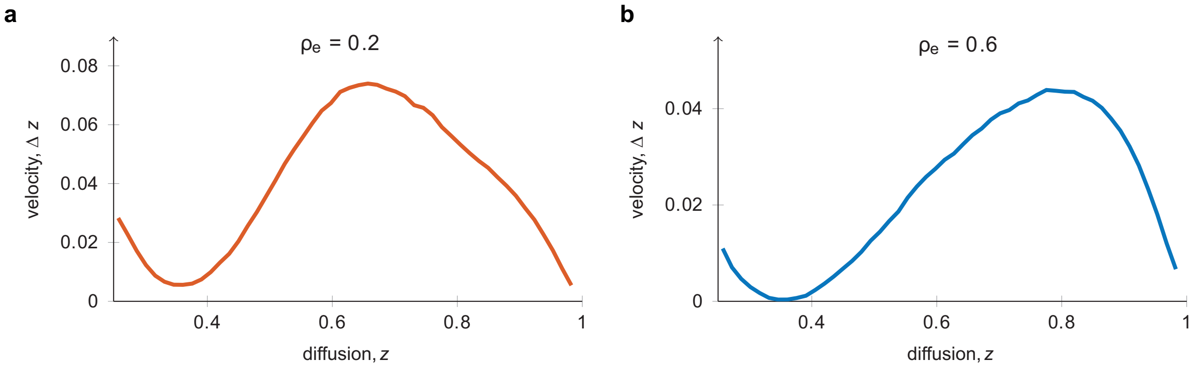

Supplementary Figure 8: **Velocity plots of the proposed model.** The results are obtained by averaging $10,000$ independent simulations. The plots show the (average) average change in the fraction of adopters of the alternative Δz as a function of the current fraction of adopters z, for two different fractions of explorers: $\rho_e = 0.2$ in panel **a** and $\rho_e = 0.6$ in panel **b**. In both plots, we set 25% of committed minority and $n = 200$ players. Both plots identify the take-off time (that is, the instant at which the diffusion takes off) close to $z = 0.4$.

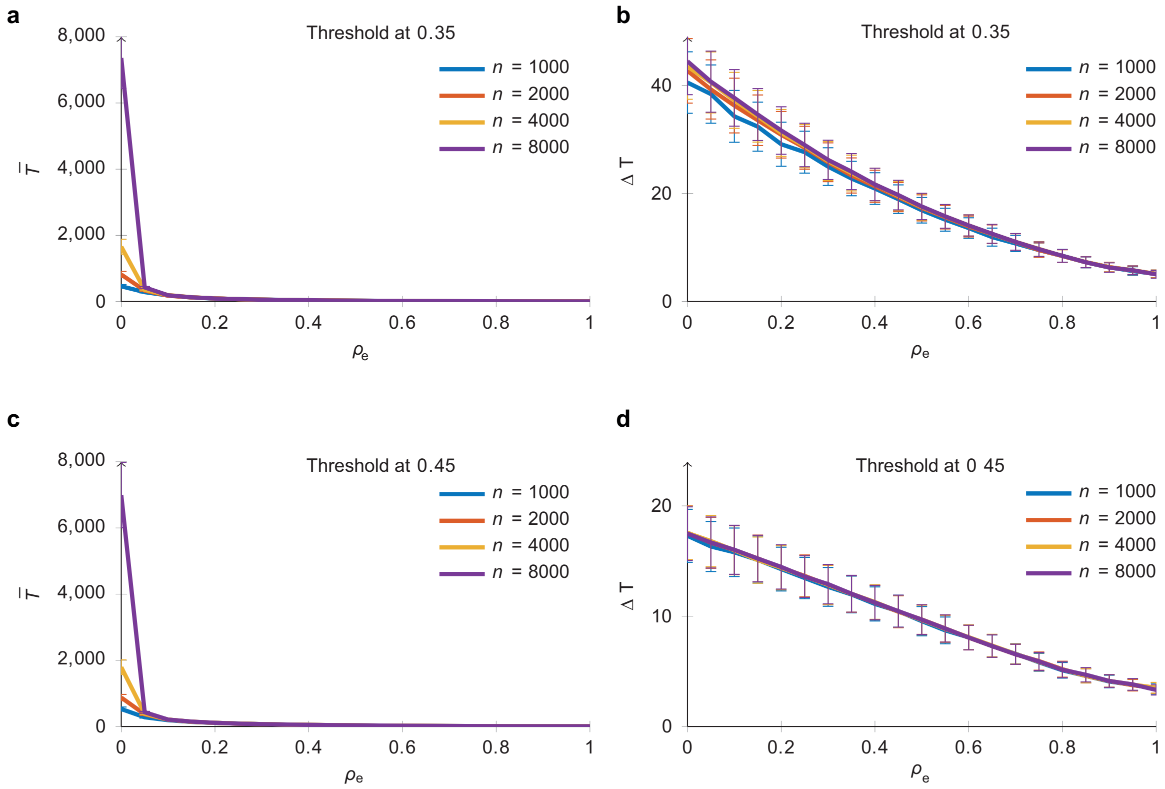

Supplementary Figure 9: **Monte Carlo simulations of the proposed model with 25% of committed minority, with different definitions of the take-off time ($\bar{T}$).** Specifically, in panels **a** and **b**, the threshold in Eq. (4b) in the main article is set to 0.35; in panels **c** and **d**, the threshold is set to 0.45. The other quantities ΔT and $\rho_{\rm e}$ are defined in the main article. The qualitative behaviour of the system is consistent with what is observed with the threshold at 0.4 (see Fig. 4 in the main article).

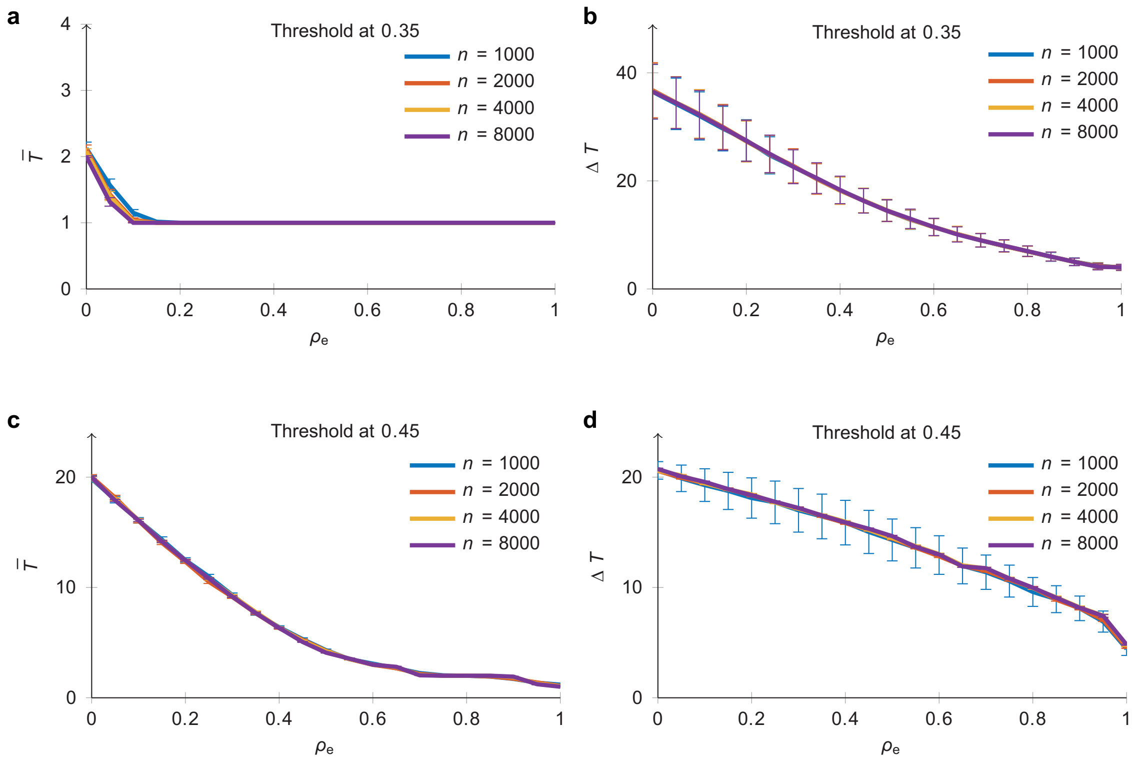

Supplementary Figure 10: **Monte Carlo simulations of the proposed model with 33% of committed minority, with different definitions of the take-off time ($\bar{T}$).** Specifically, in panels **a** and **b**, the threshold in Eq. (4b) in the main article is set to 0.35; in panels **c** and **d**, the threshold is set to 0.45. The other quantities ΔT and ρ_e are defined in the main article. In (a), we mention that setting the threshold at 0.35 with 33% of committed minority might be misleading; with 33% of the population already consistently selecting strategy 1, a threshold of 35% may be easily exceeded during the random oscillations of the stochastic process in the meta-stable state. According to $\bar{T}$ then, diffusion takeoff always occurs immediately. However, from (b)–(d), we observe that the qualitative behaviour of the system is consistent with what is observed with the threshold at 0.4 (see Supplementary Fig. 6).

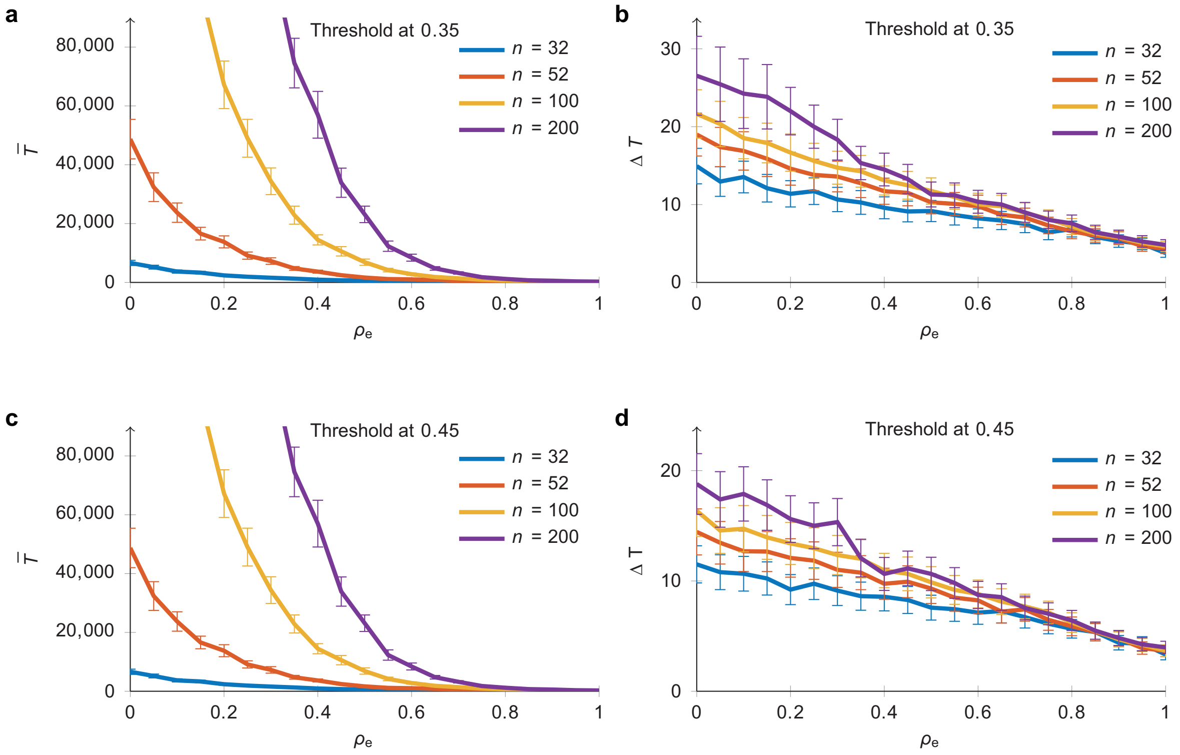

Supplementary Figure 11: **Monte Carlo simulations of the proposed model with 17% of committed minority, with different definitions of the take-off time** ($\bar{T}$). Specifically, in panels **a** and **b**, the threshold in Eq. (4b) in the main article is set to 0.35; in panels **c** and **d**, the threshold is set to 0.45. The other quantities ΔT and ρ_e are defined in the main article. The qualitative behaviour of the system is consistent with what is observed with the threshold at 0.4 (see Supplementary Fig. 5).

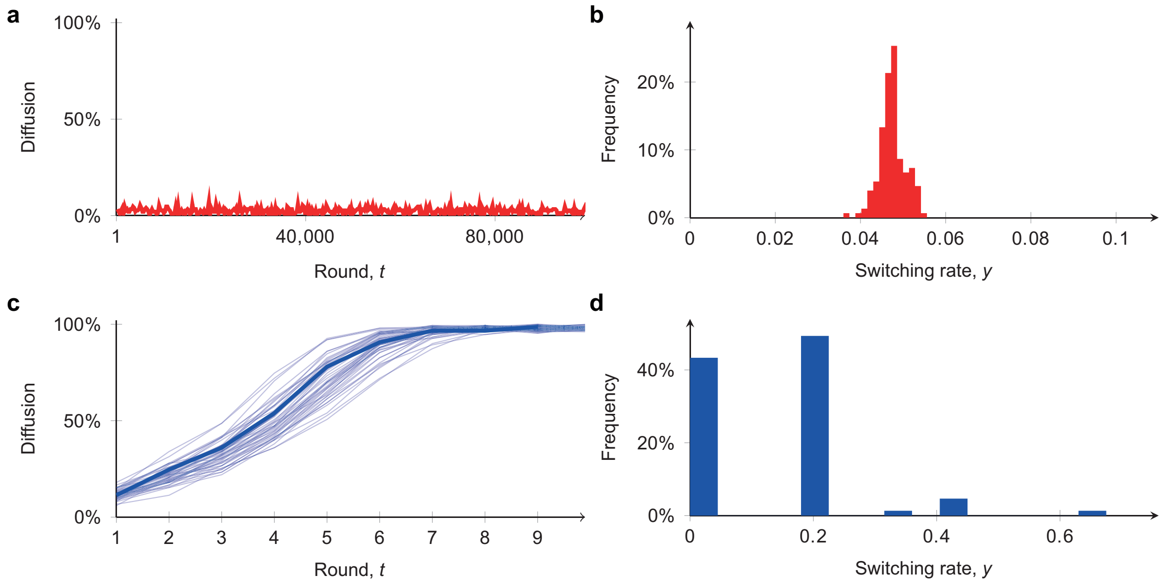

Supplementary Figure 12: **Comparison of the proposed decision-making model with a standard coordination game.** Parameters of the coordination game are $\beta_v = 4.8$ for explorers and $\beta_v = 19.7$ for non-explorers. In all figures, we set $n = 200$, 25% of committed minority, and we generate 50 independent runs of the process. One representative simulation is highlighted in each plot, with the corresponding switching rate in the right panel. **a,b** Limit case $\rho_e = 0$, in which no diffusion occurs. **c,d** Limit case $\rho_e = 1$, in which immediate diffusion is observed. In both cases, the switching rates are inconsistent with the empirical observations. This figure extends Fig. 3 in the main article.

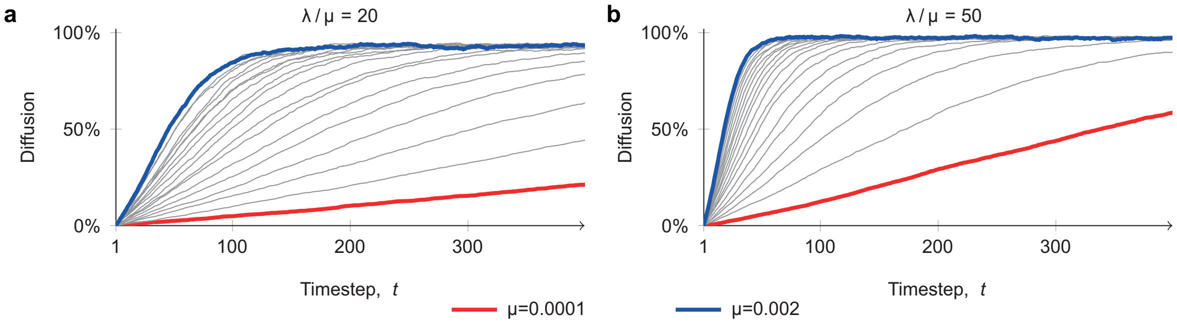

Supplementary Figure 13: **Diffusion simulated using an SIS epidemic model.** In the simulation, we set the population size $n = 200$ with 25% committed minority. All the simulations are obtained by fixing the ratio λ/μ (as detailed in the sub-caption), and varying the magnitude of μ linearly, from 0.0001 (red curve) to 0.002 (blue curve), to illustrate the range of possible behaviours that the model can reproduce. **a** The ratio between the parameters $\lambda/\mu = 20$. **b** The ratio between the parameters $\lambda/\mu = 50$.

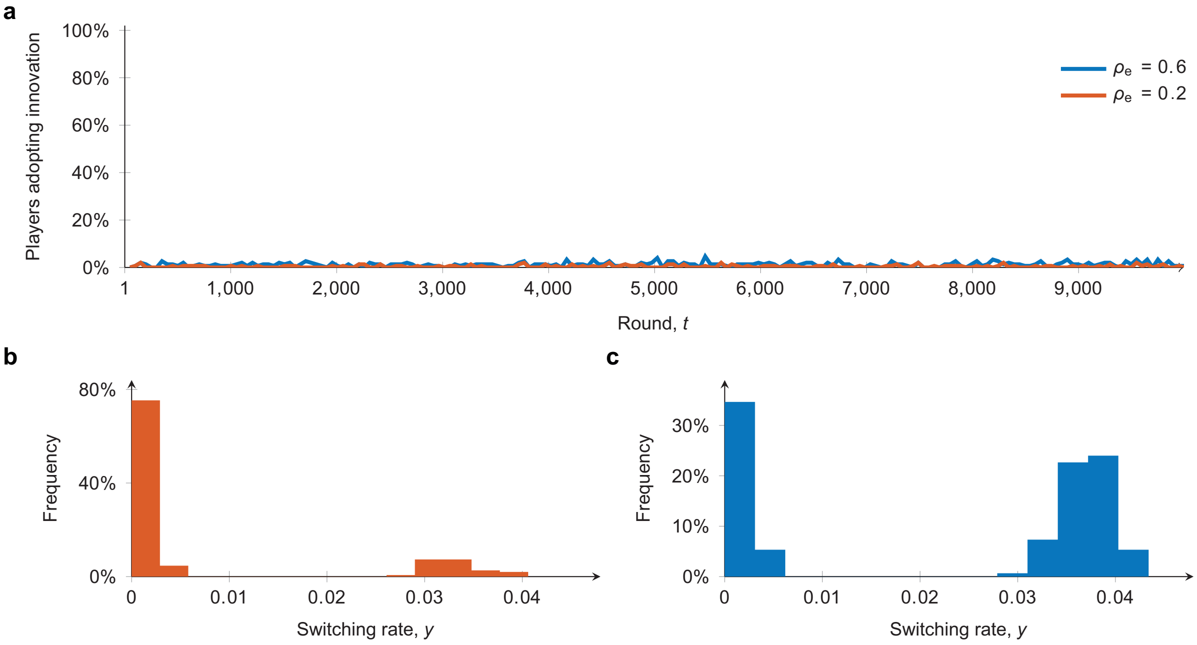

Supplementary Figure 14: **Sample simulations with only trend-seeking**. Sample simulations with $n = 200$ agents for the model with only trend-seeking (parameters in Supplementary Table 4, 25% of the population as committed minority, and two different fractions of explorers ρ_{e}; two representative simulations are highlighted. **a** Temporal evolution of the fraction of regular agents adopting the alternative. **b** Switching rates of the regular agents for $\rho_{\mathrm{e}} = 0.2$ in a representative simulation. **c** Switching rates of the regular agents for $\rho_{\mathrm{e}} = 0.6$ in a representative simulation.

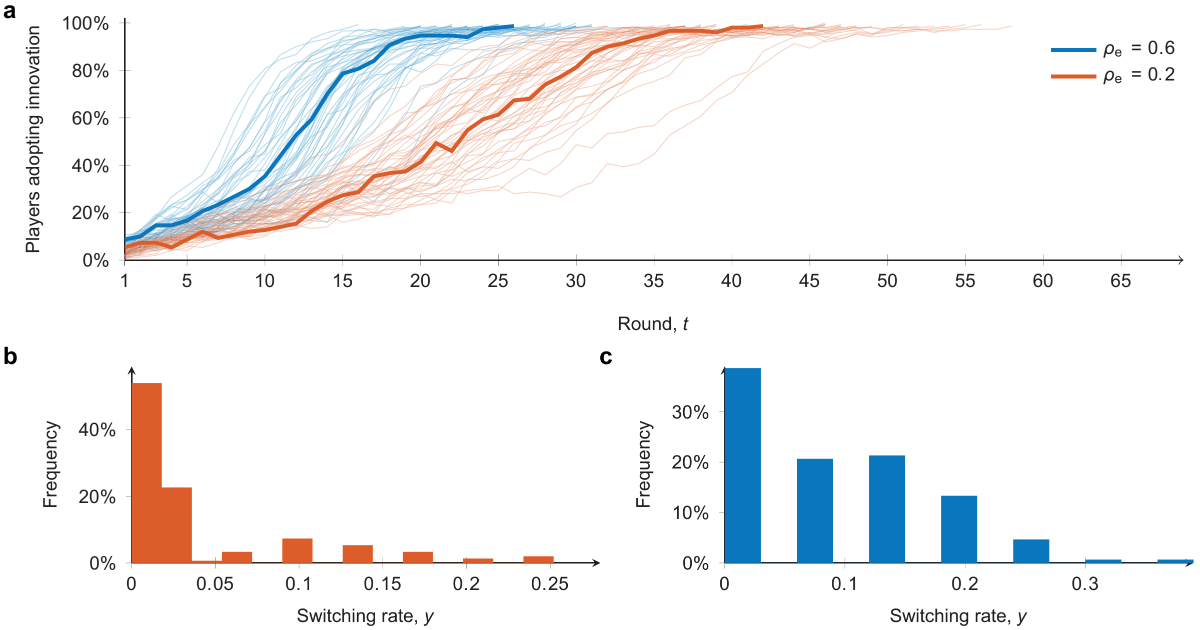

Supplementary Figure 15: **Sample simulations with only inertia**. Sample simulations with $n = 200$ agents for the model with only inertia (parameters in Supplementary Table 4, 25% of the population as committed minority, and two different fractions of explorers ρ_{e}; two representative simulations are highlighted. **a** Temporal evolution of the fraction of regular agents adopting the alternative. **b** Switching rates of the regular agents for $\rho_{\mathrm{e}} = 0.2$ in a representative simulation. **c** Switching rates of the regular agents for $\rho_{\mathrm{e}} = 0.6$ in a representative simulation.

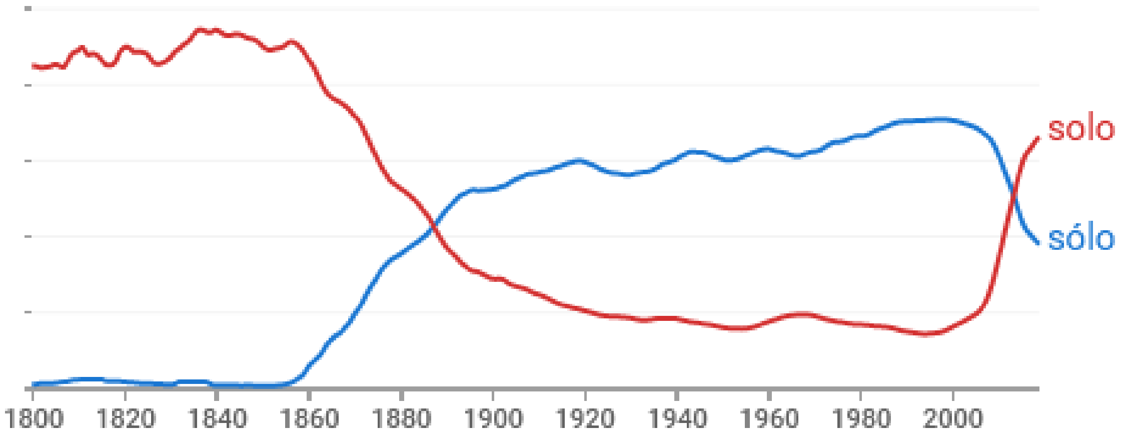

Supplementary Figure 16: **Yearly use of the spelling "sólo" and "solo" in written Spanish for the adverb that means "only".** Data are obtained from Google Ngram[18,19].

Supplementary Table 1: **Summary of the results of the experiments at the macroscopic level.** The 4 trials coloured in red are those with many irregular players (more than 33% of the group), and these trials are removed from the data used for parametrisation. In panel, it is recorded the panel of Supplementary Fig. 2 that depicts the trial. Committed minority bots is abbreviated as comm., participants who missed rounds as miss., stubborn as stub., diff. records whether social diffusion occured or not in the trial.

trial	panel	comm.	regular	dropout	miss.	stub.	diff.	rounds Phase I	$\bar{T}$	T^*	ΔT
1	a	3	6	3	0	0	no	2	-	-	-
2	b	3	6	2	1	0	no	1	-	-	-
3	c	3	6	3	0	0	yes	3	12	16	4
4	d	3	7	2	0	0	yes	2	4	7	3
5	e	3	5	3	1	0	yes	1	5	7	2
6	f	3	8	1	0	0	yes	6	11	16	5
7	g	4	5	3	0	0	yes	1	4	5	1
8	h	4	6	2	0	0	yes	2	3	6	3
9	i	4	6	0	2	0	yes	1	3	8	5
10	j	4	8	0	0	0	yes	1	2	6	4
11	k	4	7	1	0	0	yes	1	13	18	5
12	l	4	3	3	2	0	no	1	-	-	-
13	m	4	8	0	0	0	yes	2	1	5	4
14	n	2	9	0	0	1	yes	1	4	7	3
15	o	2	5	4	1	0	yes	2	4	9	5
16	p	2	8	1	0	1	yes	3	6	10	4
17	q	2	8	2	0	0	yes	2	5	9	4
18	r	2	8	1	0	1	yes	1	3	7	4
19	s	2	9	1	0	0	yes	7	9	21	12
20	t	2	10	0	0	0	no	3	-	-	-

Supplementary Table 2: **Data of experimental trial 17 and corresponding output of the classificator.** The game lasted 9 rounds, with 0 and 1 indicated selection of the status quo and alternative, respectively. If the strategy is surrounded by parenthesis, e.g. (0), this implies the strategy was selected by a computer bot (either a committed minority bot —abbreviated as comm.— or a dropout bot).

player	R1	R2	R3	R4	R5	R6	R7	R8	R9	reward	$\min_v$	maj_v	sw_v	Δ_v	output
1 (regular)	0	0	0	0	(0)	0	1	1	1	£4.50	1	0	0	1	explorer
2 (regular)	0	0	0	0	0	1	1	1	1	£4.83	1	0	0	1	explorer
3 (dropout)	(0)	(0)	(0)	(0)	(0)	(0)	(1)	(1)	(1)	-	-	-	-	-	-
4 (regular)	0	0	0	0	0	0	0	1	1	£4.17	0	1	0	-1	non-explorer
5 (dropout)	(0)	(0)	(0)	(0)	(0)	(0)	(1)	(1)	(1)	-	-	-	-	-	-
6 (regular)	1	0	0	0	1	0	1	1	1	£5.17	2	2	3	1.33	explorer
7 (regular)	0	0	0	0	0	0	1	1	1	£4.50	1	0	0	1	explorer
8 (regular)	0	0	0	0	0	0	0	0	1	£3.83	0	1	0	-1	non-explorer
9 (regular)	0	0	0	0	0	1	1	1	1	£4.83	1	0	0	1	explorer
10 (regular)	0	0	0	0	0	0	0	1	1	£4.17	0	1	0	-1	non-explorer
11 (comm.)	(1)	(1)	(1)	(1)	(1)	(1)	(1)	(1)	(1)	-	-	-	-	-	-
12 (comm.)	(1)	(1)	(1)	(1)	(1)	(1)	(1)	(1)	(1)	-	-	-	-	-	-

Supplementary Table 3: **Parameters of the model.** The parameters are obtained from the parametrisation process using the 16 reliable experimental trials (best fit and second best fit), and using all the experimental data are reported; the obtained parameters are quite robust to the inclusion/exclusion of the unreliable trials. The simulations reported in the main article are based on the best fit using 16 trials.

	β	b_e	k_e	r_e	b_f	k_f	r_f
Best fit with 16 trials	7.8	0.48	0.10	0.42	0.42	0.42	0.16
Second best fit with 16 trials	7.8	0.43	0.11	0.46	0.42	0.42	0.16
Best fit with all 20 trials	7.2	0.45	0.11	0.44	0.40	0.46	0.14

Supplementary Table 4: **Parameters of the models with only trend-seeking or only inertia.** Parameters are obtained from the parametrisation process using the experimental data.

	β	b_e	k_e	r_e	b_f	k_f	r_f
Only trend-seeking	14	0.59	0	0.41	1	0	0
Only inertia	5.8	0.96	0.04	0	0.44	0.56	0

Supplementary References

1. Wooldridge, J. M. *Econometric Analysis of Cross Section and Panel Data* (MIT Press, Cambridge MA, US, 2010), 2 edn.

2. Ross, S. M. *Introductory Statistics* (Academic Press, Cambridge MA, US, 2017), 4 edn.

3. Samuelson, W. & Zeckhauser, R. Status Quo Bias in Decision Making. *Journal of Risk and Uncertainty* **1**, 7–59 (1988).

4. Leary, M. R. & Kowalski, R. M. Impression Management: A Literature Review and Two-Component Model. *Psychological Bulletin* **107**, 34 (1990).

5. Sparkman, G. & Walton, G. M. Dynamic Norms Promote Sustainable Behavior, Even If It Is Counternormative. *Psychological Science* **28**, 1663–1674 (2017).

6. Noelle-Neumann, E. *The Spiral of Silence: Public Opinion, Our Social Skin* (University of Chicago Press, 1993).

7. Mortensen, C. R. *et al.* Trending norms: A lever for encouraging behaviors performed by the minority. *Social Psychological and Personality Science* **10**, 201–210 (2019).

8. Centola, D., Becker, J., Brackbill, D. & Baronchelli, A. Experimental evidence for tipping points in social convention. *Science* **360**, 1116–1119 (2018).

9. Bettencourt, L. M., Cintrón-Arias, A., Kaiser, D. I. & Castillo-Chávez, C. The power of a good idea: Quantitative modeling of the spread of ideas from epidemiological models. *Physica A: Statistical Mechanics and its Applications* **364**, 513 – 536 (2006).

10. Granovetter, M. Threshold Models of Collective Behavior. *American Journal of Sociology* **83**, 1420–1443 (1978).

11. Kempe, D., Kleinberg, J. & Tardos, E. Maximizing the Spread of Influence through a Social Network. In *Proceedings of the 9th ACM SIGKDD International Conference on Knowledge Discovery and Data Mining*, 137–146 (2003).

12. Brown, M. J. & Satterthwaite-Phillips, D. Economic correlates of footbinding: Implications for the importance of Chinese daughters' labor. *PloS One* **13**, e0201337 (2018).

13. Kroch, A. S. Function and grammar in the history of English: Periphrastic do. In *Language Change and Variation*, 133–172 (John Benjamins Publishing Company, Amsterdam, The Netherlands, 1989).

14. Amato, R., Lacasa, L., Díaz-Guilera, A. & Baronchelli, A. The dynamics of norm change in the cultural evolution of language. *Proceedings of the National Academy of Sciences* **115**, 8260–8265 (2018).

15. Mackie, G. Ending footbinding and Infibulation: A Convention Account. *American Sociological Review* 999–1017 (1996).

16. Denison, D. *English Historical Syntax* (Routledge, Abingdon-on-Thames, UK, 1993), 1 edn.

17. Vulanović, R. Fitting periphrastic do in affirmative declaratives. *Journal of Quantitative Linguistics* **14**, 111–126 (2007).

18. Michel, J.-B. *et al.* Quantitative analysis of culture using millions of digitized books. *Science* **331**, 176–182 (2011).

19. Google. Books ngram viewer. `https://books.google.com/ngrams`. Accessed: 27 April, 2021.

20. Ye, M. *et al.* Collective patterns of social diffusion are shaped by individual inertia and trend-seeking. *Zenodo* (2021). DOI: 10.5281/zenodo.5175151.
